# Noninvasive assessment of autonomic modulation of heart rate variability in the Ts65Dn mouse model of Down syndrome: A proof of principle study

**DOI:** 10.14814/phy2.14486

**Published:** 2020-06-19

**Authors:** Adriano L. Roque, Mark W. Johnson, Melissa R. Stasko, Luiz C. de Abreu, Talita D. da Silva, Alberto C.S. Costa

**Affiliations:** ^1^ Division of Pediatric Neurology Department of Pediatrics Case Western Reserve University Cleveland OH USA; ^2^ Postgraduate Program in Medicine, Cardiology Federal University of Sao Paulo Sao Paulo SP Brazil; ^3^ Design of Studies and Scientific Writing Laboratory in the ABC School of Medicine Sao Paulo Brazil; ^4^ Department of Psychiatry Case Western Reserve University Cleveland OH USA

**Keywords:** animal models, autonomic nervous system, cardiovascular system, Down syndrome, heart rate, Ts65Dn

## Abstract

**Introduction:**

The Ts65Dn mouse is the most widely used animal model of Down syndrome (DS). Differences in autonomic regulation of heart rate variability (HRV) in individuals with DS have been hypothesized. Pharmacological studies in animal models may help us understand mechanisms underlying observed changes in HRV in people with DS.

**Objective:**

To investigate the use a new, noninvasive technique to assess cardiac autonomic modulation in Ts65Dn mice under the effect of adrenergic and cholinergic agonists.

**Method:**

We recorded electrocardiograms (ECGs) from 12 Ts65Dn and 12 euploid control mice. A 30‐min baseline recording was followed by the injection of an adrenergic (isoproterenol [Iso]) or cholinergic (carbachol [CCh]) agonist. Heart rate and HRV were analyzed using a series of methods customized for mice.

**Results and Discussion:**

The ECG apparatus described here allowed us to detect noninvasively long series of heartbeats in freely‐moving animals. During baseline conditions, the yield of detectable heartbeats was 3%–27% of the estimated total number of events, which increased to 35%–70% during the 15‐min period after either Iso or CCh injections. Ts65Dn mice displayed a robust enhanced Iso‐induced negative chronotropic rebound response compared with euploid control mice. We observed a significantly smaller CCh response in Ts65Dn versus control euploid mice in the 6‐ to 10‐min‐interval postcarbachol injection.

**Conclusion:**

This work showed that the techniques described here are sufficient for this type of study. However, future studies involving the use of more selective pharmacological agents and/or genetic manipulations will be key to advance a mechanistic understanding of cardiac autonomic regulation in DS.

## Graphical abstract

We used a new, noninvasive technique to assess cardiac autonomic modulation in the Ts65Dn mouse model of Down syndrome under the effect of adrenergic and cholinergic agonists. This new ECG recording apparatus has the potential to be very useful to investigators studying heart rate and heart rate variability in rodent models of human disorders. However, future studies involving the use of more selective pharmacological agents and/or genetic manipulations of the mouse models of DS will be necessary to complement this work.

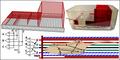

## 1 INTRODUCTION

Down syndrome (DS), the most common genetic cause of intellectual disability, occurs in about 1 per 700 births (Parker et al., 2010). In addition to trisomy 21, two other forms of DS are known, translocation and mosaicism (Patterson & Costa, 2005). Several comorbidities are associated with DS, including congenital heart and gastrointestinal malformations, acute childhood leukemia, hypothyroidism, and seizure disorders (Roizen & Patterson, 2003; Weijerman & de Winter, 2010).

In the early 1990s, the first postnatal viable mouse model for DS, the Ts65Dn mouse, was created by Dr. Muriel Davisson and her team (Davisson & Costa, 1999; Davisson, Schmidt, & Akeson, 1990). In subsequent years, with the advent of chromosomal engineering, several other mouse models have been described for DS studies (Costa & Scott‐McKean, 2013; Herault et al., 2017; Liu et al., 2011). The Ts65Dn mouse, however, is still the only widely available animal model of DS with a free trisomy. The Ts65Dn extra chromosome encompasses most of the human chromosome 21 orthologous region of mouse chromosome 16 (Costa & Scott‐McKean, 2013; Davisson & Costa, 1999; Herault et al., 2017), but also include a small, gene‐rich region of mouse chromosome 17 that is not orthologous to human chromosome 21 (Duchon et al., 2011).

Most of the research involving Ts65Dn mice has focused on the central nervous system. However, there have also been some studies aimed at characterizing cardiovascular phenotypes in Ts65Dn mice, which have been primarily directed at congenital heart malformations (Li et al., 2016; Williams, Mjaatvedt, & Moore, 2008). Many types of cardiac malformations have been described in these mice, such as right aortic arch with Kommerell's diverticulum, persistent truncus arteriosus, abnormal right aortic arch with right‐sided ductus arteriosus, and aberrant right subclavian artery (Williams et al., 2008).

Recently, DeRuisseau, Receno, Heffernan, and Cunningham (2019) published an investigation of heart rate and blood pressure in Ts65Dn mice. These authors observed lower mean blood pressure in Ts65Dn mice compared with euploid control mice, which they attributed to potential genotype‐dependent autonomic differences in these animals. This particular finding concurred in part with human studies showing low heart work capacity, chronotropic incompetence, and significant reductions in basal blood pressure and heart rate and blood pressure responses to exercise in persons with DS (Baynard, Goulopoulou, Sosnoff, Fernhall, & Kanaley, 2014; Fernhall & Otterstetter, 2003; Fernhall et al., 1996, 2001; Figueroa et al., 2005; Guerra, Llorens, & Fernhall, 2003; Santoro et al., 2020). The hypothesis advanced by most investigators in the field is that the differences observed in cardiovascular physiology in persons with DS are due to cardiac autonomic dysfunction (Fernhall, Mendonca, & Baynard, 2013). For example, Iellamo et al. (2005) suggested that such dysfunction results from a decrease in both sympathetic and vagal activation.

The issue of potential differences in autonomic regulation in individuals with DS has also been invoked repeatedly in the study of heart rate variability (HRV) in individuals with DS. Currently, HRV primarily is viewed as a noninvasive method to analyze the parasympathetic modulation of heart rate (da Silva et al., 2018). However, the low‐frequency (LF) band is believed to contain both parasympathetic and sympathetic influences, which makes it a difficult band to interpret, with some research even suggesting the LF is a better marker of baroreflex sensitivity (Bonyhay, Risk, & Freeman, 2013; Cygankiewicz & Zareba, 2013). HRV has been used to study mechanisms related to cardiac autonomic modulation in both physiological processes and pathologic conditions (Campen, Tagaito, Jenkins, Balbir, & O'Donnell, 2005; Vanderlei, Pastre, Hoshi, Carvalho, & Godoy, 2009). However, despite a substantial number of studies looking at HRV indices in people with DS, the relationship between HRV and DS is still poorly understood.

de Carvalho et al. (2015) showed that children with DS exhibit increase of sympathetic and global modulation indices (Carvalho et al., 2018). More recently, Carvalho et al. presented a meta‐analysis and systematic review of HRV in people with DS. The 13 articles used in the study did point toward autonomic dysfunction in people with DS. However, the exact nature of such dysfunction and its physiological mechanisms remain unclear (Carvalho et al., 2018).

Several studies in mice have used HRV to analyze various physiologic or pathologic processes (Campen et al., 2005; Liu, Wei, Kuang, Tsien, & Zhao, 2014; Park et al., 2009). However, there have been no specific reports on HRV in Ts65Dn mice, with the exception of an abbreviated heart rate spectral analysis performed by DeRuisseau et al. (2019). Therefore, in this study, we report data obtained through the use of a new noninvasive electrocardiogram (ECG) recording technique to investigate HRV in Ts65Dn mice, as an attempt to explore whether these animals replicate the autonomic dysfunction supposedly associated with DS. One of the clear advantages of using an animal model in this context is that we can use pharmacological means to specifically stimulate the adrenergic or cholinergic system.

The objectives of this proof‐of‐principle investigation are (a) to establish the usefulness of a new, noninvasive, long‐term recording technique, and (b) to shed some light on the mechanism underlying the observed changes in HRV presented in people with DS. In other words, instead of being a search for definitive answers, the overarching goal of this paper was to introduce a new technique and inform the rational design of future studies in both murine models and in individuals with DS.

## 2 MATERIALS AND METHODS

### 2.1 Study design

This was a repeated measures descriptive study, conducted at the Case Western Reserve University (CWRU). Cardiac data collection was performed with a noninvasive rack installed in a standard mouse cage, which sensed ECG through the feet of freely moving mice. The ECG was used for the analysis of HRV.

### 2.2 Animals

All experimental procedures were approved by the CWRU Institutional Animal Care and Use Committee, and performed in the CWRU Animal Resource Center facilities. Adult male Ts65Dn and euploid control mice, aged 4–7 months, were generated by in‐house repeated backcrossing (>20 generations) of Ts65Dn females (originally purchased from JAX, Bar Harbor, ME; Stock No. 005252) with B6EiC3Sn.BLiAF1/J hybrid male mice (C57BL/6JEiJ females × C3Sn.BLiAmales, also from JAX). Female mice were excluded from this study because the Ts65Dn females were retained for breeding, given that the Ts65Dn male mice of this strain have close‐to‐null fertility. Mice were housed together with littermates, with ad libtum access to food and water, at normal room temperature, on a 12:12h light–dark cycle (lights on at 6 a.m.). Recordings were performed during daytime hours with the lights on and no access to food or water during the 2‐hr recordings. A total of 12 Ts65Dn and 12 euploid controls were included in this study. Many mice were littermates. The age range was 113–206 days for both groups. Control and Ts65Dn average ages and weights were 152 and 158 days, and 34.4 g (22.9–42.9) and 26.0 g (20.5–41.2), respectively.

### 2.3 Cardiac recordings

**FIGURE 1:**
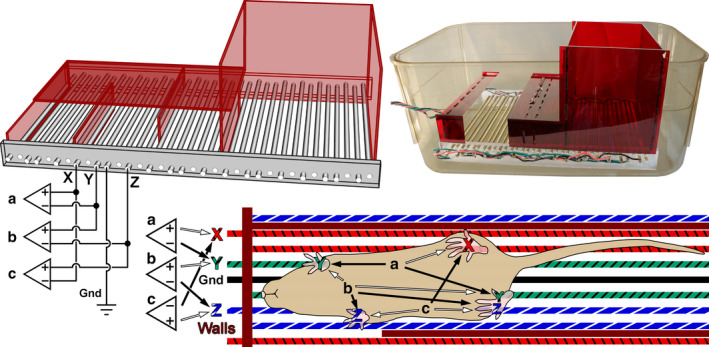
Cutaway drawing and photographic illustration of electrocardiograms (ECG) apparatus. The lower diagram illustrates arbitrary foot placement on the bars of the apparatus, and how bar sets X, Y, and Z are differentially amplified to give three ECG signals (A, B, and C)

During each recording session, one mouse was removed from its cage and placed into a clean cage containing an ECG recording device (rack) developed in our laboratory. The ECG rack consisted of a series of closely spaced parallel metal bars mounted above the floor of the cage. The bars were electrically connected into six distinct sets plus a seventh grounding set, with five bars in each set, and arranged in an interlaced pattern so that at any moment the mouse would be likely to have its feet resting on bars from more than one group (see Figure 1). External to the cage, a set of toggle switches combined the six sets into three groups plus the ground (X,Y,Z,G). The toggle switches allow reassignment of the bars into various patterns (such as XYZGXYZ or XXYGYZZ). The three pairs of bars groups (X‐Y, Y‐Z, Z‐X) were differentially amplified using three instrumentation amplifiers (A,B,C) based on the AD8232 “Single‐Lead, Heart Rate Monitor Front End” (Analog Devices, Norwood, MA ‐ USA), configured to a 1–168 Hz bandwidth. This device features a design that does not require a third lead to each amplifier, and recovers rapidly after electrode disconnect‐reconnect. Signals were acquired at 16‐bits and 5 kHz using a Digidata 1322A digitizer (Molecular Devices, LLC., San Jose, CA, USA). The rack included a dark‐red plexiglas chambered cover that was designed to provide an optional darkened environment which encouraged the mice to stay on all four feet with left and right limbs on different bar groups. Examples of when ECG signal were very poor included when the mouse: (a) sat on its hind limbs and groomed itself; (b) continuously moved around the cage; or (c) laid down on the bars with its feet hanging through. Technically, the mouse tail and snout, as well as its four feet all provided electrical tap points into the ECG. Thus, as the mouse moved around the cage, the best ECG signal from the amplifiers frequently switched among the three recorded channels.

For this study, two sessions of ECG spaced several days apart were recorded and analyzed for each mouse. During the first session, the mouse was given an intraperitoneal (i.p.) injection of 0.2 mg/kg of the adrenergic agent isoproterenol (Iso). During the second session, the mouse was given an i.p. injection of 0.2 mg/kg of the cholinergic agent carbachol (CCh). For each session, the mouse was transferred onto the rack in a clean cage and the ECG recording was started. At approximately 29 min of elapsed time, the mouse was removed from the cage to administer the injection, then immediately returned to the cage, where recording continued for a total of 2 hr. The actual time of injection was recorded as accurately as possible, so that drug response timing could be aligned across all mice. Since the mice were allowed to free roam during the recording, there was a great deal of variability as to which rack bars their feet were touching. Throughout the recording, if the mouse was sitting still but signals were sub‐optimal, the toggle switches were flipped to a different bar configuration in order to improve the ECG quality.

The ECG signals were analyzed to extract RR intervals (RRI) using custom algorithms written in the Igor Pro software analysis platform (Wavemetrics, Inc. Portland, Oregon—USA). Because of the free mouse movement, a large part of the software was dedicated to artifact rejection and determining which channel was optimal at a given moment. The three channels were each preprocessed similarly, but independently, and then RRI were jointly extracted from all three channels. Processing steps included: (a) smoothing, bandpass and notch filtering the ECG; (b) use of the Hilbert transform to impart a 90° phase shift to the signal; (c) combination of each ECG signal together with its Hilbert transform into a complex‐valued stream, where the magnitude of the complex value (distance from 0,0) at each time point provided a good proxy of ECG amplitude relatively independently of the shape of ECG waveform; and (d) the magnitude was used to construct smoothed middle, lower, and upper envelopes, which were in turn used to adjust and normalize the signals.

For R‐wave time detection, the complex‐valued ECG signals were cross‐correlated with one or more complex‐valued reference signals, then normalized using the envelope signals to produce a magnitude signal that typically varied between zero and one, where the peak was maximum at the time when the ECG best matched the reference signals and where the larger the amplitude, the better the ECG matched the reference. These signals provided three simultaneous channels of beat‐matching information, where the “best” channel(s) could be selected based on the amplitude. R‐wave times were determined for each channel. The highest quality channel was used to select the time, but if multiple channels were nearly the same quality, the same channel was utilized throughout that segment, rather than bouncing back and forth between channels. If no channels were of sufficient quality, the time was marked as unreliable and removed from the RR analysis. Because one of the best predictors of the next RRI is the nearby neighbors, the process of detecting R‐wave times and RRI was iterative, using median smoothed RRIs and RR variability estimates from prior iterations to estimate the next likely RRI range. This was critical for the rapidly changing mouse HR, where the beat‐to‐beat change frequently exceeds 25 ms and minute‐to‐minute change exceeded 100 ms.

### 2.4 HRV analysis

Heart rate variability was analyzed with custom algorithms written in the IgorPro using nonconventional metrics. Conventional measures used in human HRV are not scaled for mouse ECG. Typically, human ECG analysis separates HRV components into high frequency (HF, 0.15–0.4 Hz or 2.5–6.6 s periods); low frequency (LF, 0.04–0.15 Hz or 6.6–25 s); and sometimes very low and ultra‐low frequencies (VLF, UFL) (Shaffer & Ginsberg, 2017).

Thireau, Zhang, Poisson, and Babuty (2008) reviewed methods published by a number of authors and laboratories for analyzing mouse ECG. Most mouse ECG analyses multiply these frequencies by approximately 10‐fold, which is not unreasonable, since the mouse HR and respiration are about 10 times faster than what is typically seen in human beings. However, HRV analyses generally ignore the fact that the primary driving influence of the HF component is respiratory coupling, and respiration rate varies quite widely in both human and mouse studies.

Most studies also ignore the fact that the VLF and ULF components are strongly influenced by external factors such as exercise, sleep (REM and non‐REM), stress, pharmacological interventions, and many other factors. In our study, we used nonlinear methods to split the RRIs into three components; a fast component that is primarily respiratory coupled (SD‐fast); a very slow component (RR‐vslow) that gives HR trends due to our pharmacological interventions or behavioral changes, and a moderately slow component that falls in the middle (SD‐slow). The three components summed together equal the original RRI sequence (RR‐orig). Because our data has a wide range of signal quality, all analyses required robust methods that tolerate dropped sections of data. The high‐frequency component, SD‐fast, is closely related to the conventional successive differences (SD) metric, but we observed that it more directly tracks the respiratory coupled HRV component. The first step calculates the median of a few nearest‐neighbor RRIs (±4 in our analysis), excluding the RRI itself. The median is subtracted from each RRI giving a signal similar to the SD metric, but that maintains the shape of the underlying fast variation. The SD‐fast component is subtracted from RR‐orig sequence, giving a greatly smoothed sequence. Most conventional smoothing or filtering algorithms suffer badly when attempting to remove this highly variable HRV component. This median based algorithm does remarkably well, but leaves a small component of high‐frequency residual in the RR sequence. A short box smoother (seven beats in our analysis) effectively removed most of the residual rapid variation, after which the SD‐fast component was recalculated by subtracting this double smoothed RR from the original RR. The second step calculated RR‐vslow by taking the smoothed RR and applying a hybrid median and box smoother, which averaged the middle 50th percentile of RRIs over a much wider range (±100 beats in our analysis). Finally, the mid‐frequency component SD‐slow was calculated as:

SD-slow=RR - orig - SD - fast - RR - vslow

Both SD‐fast and SD‐slow are nominally zero‐centered, so the root‐mean‐square (RMS) over short or longer intervals gives measures of fast and slow variability magnitude. RR‐vslow encompasses the heart rate trend changes. Two measures, peak and/or mean value, were used to quantify the heart rate baseline and changes in response to pharmacological or other influences.

### 2.5 Analysis periods

The time course of Iso and CCh injections are rather different, so analysis periods were chosen separately for the two drugs. During the baseline period, the mouse activity level varied widely (exploring, grooming, sleeping, etc.). By comparison, after injection of either Iso or CCh the activity level was greatly curtailed for 30–60 min, with the resultant effect being a much higher yield of good ECG and RRIs during this recovery period. For all analyses, the means of RR‐orig and RR‐vslow, and the RMS of SD‐slow and SD‐fast were calculated using all valid RRIs in each 15‐s epoch. Baselines were averaged over each epoch from the entire 29‐min period prior to injection. The peak Iso response was taken as the minimum RR‐orig epoch (fastest HR) over the 1.25 min following injection (Iso 1). The effect of the Iso rapidly diminished over several minutes. RRI measures were calculated for the plateau of the recovery period, 10 to 15 min after injection (Iso 10–15). For CCh injections, the RRI rapidly increased (HR slowed) reaching a plateau within approximately 2 min. RRI measures were calculated for the postinjection periods of 2–6 min (maximal slowing, CCh 2–6) and 6–10 min (partial recovery, CCh 6–10) period.

### 2.6 Statistics

Two‐tailed, unpaired, unequal variance *t*‐tests were used to compare HRV parameters between Ts65Dn and control groups, at each of the analysis periods. Two‐tailed, paired, unequal variance *t*‐tests were used to assess whether the response to Iso and CCh were statistically different from baseline within each group. For all comparisons, a “*p*” value of <.05 was selected as the criterion for statistical significance.

## 3 RESULTS

Overall, our results demonstrate the ability to successfully recording long stretches of ECG events through the feet of Ts65Dn mice and their control siblings using the new apparatus described here. The methods worked well to get ECG traces and RRI measures throughout the 2‐hr recording period, but the yield (estimated percentage of good RRIs measured) varied widely, depending on mouse behavior. During each epoch in the baseline period, an estimated 3% and 27% of the beats were detected. In contrast, during the 15‐min period after both Iso and CCh injections, the yield was much higher, ranging from about 35%–70%. All data segments where several successive beats could not be detected reliably were excluded. The supplementary information section gives additional details on the yield and gives a detailed example of detection during a slightly noisy ECG segment.

**FIGURE 2:**
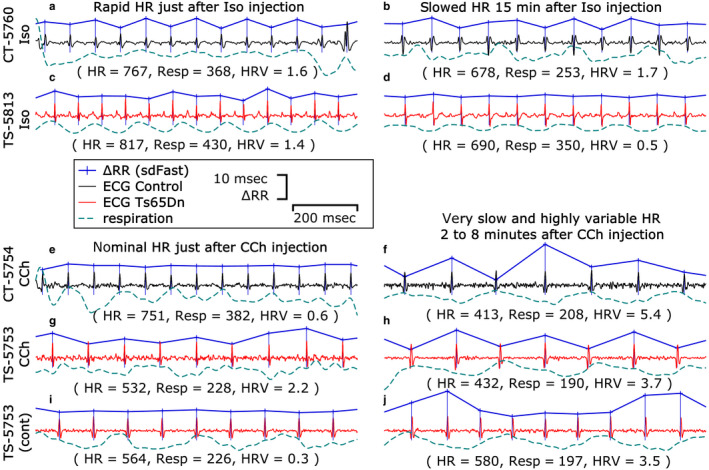
Sample electrocardiograms (ECG) traces from control mice (a,b,e,f in black) and Ts65Dn mice (c,d,g–j in red), with respiration underneath ECG (low pass filtered from ECG), and the detected ΔRRIs (SD‐fast component shown above ECG). The upper groups (a–d) were given Iso injections, and the lower groups (e–j) were given CCh injections. The left‐hand panel times (a,c,e,g) were just after injection, and the right‐hand panel times (b,d,f,h) were from the plateau periods. i and j show two additional atypical examples from mouse TS‐5753. Below each set of sample traces are the average heart and respiration rates (HR, Resp), and heart rate variability measure (HRV, standard deviation of SD‐fast) for the brief periods shown. These examples were chosen because they all show fair to excellent respiration signal, however, the majority of the time no clear respiration signal is apparent. All the mice in this study commonly had a respiration rate that was approximately ½ of the ECG rate as evidenced by alternating beat‐to‐beat RRIs (saw tooth pattern, a,b,f,g,h). Less commonly, the ratio is between 2–3 beats per breath. The HR escalation due to Iso injection (a = 767 bpm, c = 817 bpm) was very short in duration, lasting 1–2 min, with a more extended slowing phase lasting 15–30 min (b = 678 bpm, d = 690 bpm). The effects of the CCh injection were slower (e = 751 bpm, g = 532 bpm just after injection), with a rapidly slowing HR and increase in HRV occurring over 30–120 s (f = 413 bpm, h = 432 bpm), with sustained slowing, returning halfway back to baseline within approximately 7–14 min. The segment in i is atypical because the SD‐fast component was very small for approximately 30 s at 13.25 min after CCh injection (see Figure 4d). The segment in j is atypical because of sustained rapid HRV that was slower than the respiration rate for approximately 106 s starting at 13.75 min after CCh injection (see Figure 4d). The period of HR oscillation started at 0.60 s (100 per minute or every 5.7 beats at 105 ms RRI) slowing to 0.82 s (73 per minute or every 8.3 beats at 99 ms RRI). By comparison, the respiration rate in j is every three beats

Figure 2 shows detailed 1‐s sample traces for both Ts65Dn and control mice, illustrating the effects of Iso and CCh on the RRIs and HRV. A very fast respiratory coupled HRV component is prevalent throughout all the recordings, during baseline, after Iso and CCh injections, and during and after recovery from the injections. Occasionally, a well‐discernible respiratory signal can be extracted from the ECG signal. The examples in Figure 2 were selected for their respiratory signal, which, when present, is normally in lock‐step with the HRV. Figure 2j is an atypical exception.

**FIGURE 3:**
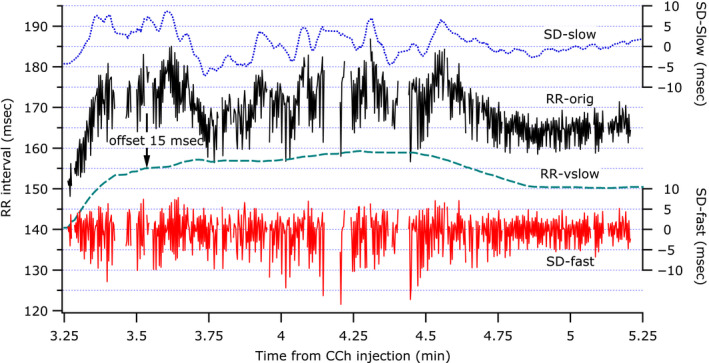
A 2‐min example of the different RRI components shortly after CCh injection from a study where all three split components were visibly present. From top to bottom the traces are SD‐slow, RR‐orig, RR‐vslow offset down by 15 ms for clarity, and SD‐fast. SD‐slow and SD‐fast are zero‐centered and shown on right‐hand axes at the same vertical scale as the absolute RRIs shown on the left‐hand axis. This example illustrates the drastic time‐scale differences of the RR components, yet comparable amplitude fluctuations

Figure 3 shows a 2‐min example from one mouse, illustrating the separation of the RRIs from the RR‐orig into the RR‐vslow, SD‐slow, and SD‐fast components. Data displayed in 2‐min sweeps are generally too dense to resolve the individual saw‐toothed patterns of the SD‐fast component, but the fluctuating band of points is quite apparent here. This example also has a strong SD‐slow component, which is very distinct in its nature from SD‐fast. The RR‐vslow component shows the background trend with the other two components removed. The 2‐min example is a little too short to show the full trend caused by the CCh injection.

**FIGURE 4:**
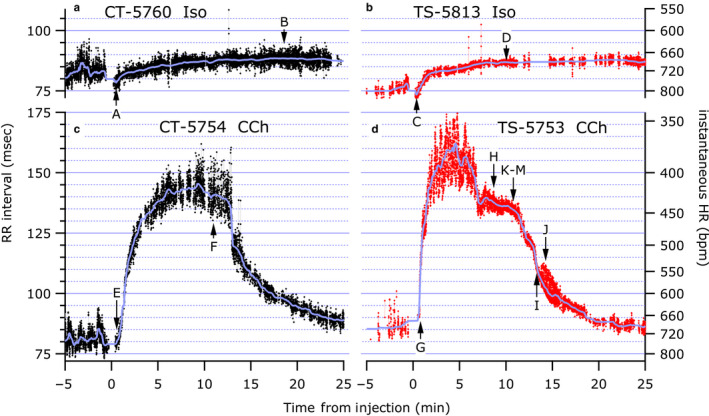
Thirty‐minute example of all valid RRIs from four recordings (control mice a & c on the left in black; Ts65Dn mice b & d on the right in red) overlaid by the RR‐vslow component. The upper group (a, b) had Iso injections, and the lower group (c, d) had CCh injections. The time axis shows 5 min of baseline data. The injections were given at time 0. The arrows labeled A–M show time points of the detailed strips shown in Figure 2 and S2

Figure 4 shows 30‐min examples from Ts65Dn and Control mice in response to Iso and CCh injections. Automatic computerized detection and rejection of good and bad RRIs works fairly well, but these examples were manually reviewed to verify and correct or eliminate suspicious or anomalous RRIs. Thus, these examples are clean. As such, in 3A, for example, two brief bradycardic events are visible, and three are visible in 3B. There are clearly some larger sections of poor signal where RRIs are dropped for an extended period, but many of the dropped periods are too brief to be resolved at this time scale. Here again, except where noted in 3D at arrow J, the majority of the high‐frequency variability is respiratory coupled, and usually at about two heart beats per breath.

**FIGURE 5:**
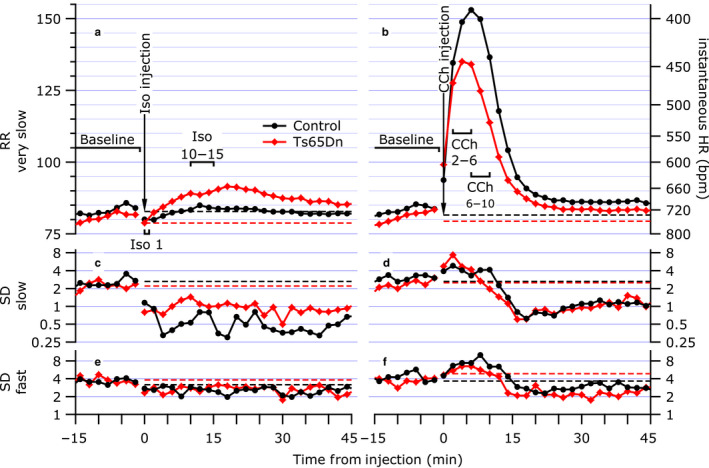
Ensemble averages at each 120 s epoch from 15 min before injection to 45 min after injection of a & b RR‐vslow (RRI and IHR scales), and ensemble standard deviations of c & d SD‐slow and e & f) SD‐fast (log scales) from 12 control mice (black with circles) and 12 Ts65Dn mice (red with diamonds). The left (a, c, e) and right hand (b, d, f) panels are for Iso and CCh injection, respectively. The primary analysis periods indicated by the brackets are as follows: baseline for both agents (start of study to removal for injection); Iso 1—peak HR just after injection; Iso 10‐15—plateau period during recovery; CCh 2‐6—maximal slowing response period; CCh 6‐10—later period during partial recovery from CCh. The dashed line indicates the baseline averages for each group and measure

Figure 5 shows ensemble averages of all the recordings analyzed in this study (12 each: Control Iso; Ts65Dn Iso; Control CCh; and Ts65Dn CCh). In this example, longer epochs (120 s) were used to show smoother curves. Most results summarized in Table 1 are readily apparent in this figure. At Iso 1, the brief increase in HR (low RRI) is visible, but the difference between Ts65Dn and Controls is not statistically significant. However, during the recovery period, at Iso 10–15, the Ts65Dn group was significantly slower than the Controls and baseline. There were no significant differences in the RMS of SD‐fast during this time period. Regarding the CCh injections, both the CCh 2‐6 and the CCh 6‐10 were highly significantly different from baseline for RR‐orig, but only CCh 6‐10 showed a difference between the groups. (That was primarily because the Ts65Dn mice appeared to have a shorter recovery time on average.) The SD‐fast component increased significantly during the slow RR period, without any group differences, then rebounded lower than baseline in the period beyond 15 min, continuing to remain low for a substantial period of time. The SD‐slow component showed a similar shape profile to that of SD‐fast, but only the Ts65Dn group in CCh 6‐10 was significantly different from baseline.

**TABLE 1:** Statistical results summary

Genotype	Time period	Description	RR‐orig		SD‐slow		SD‐fast	
			(ms)	Equiv. IHR (bpm)	Log (RMS) log (ms)	Equiv. RR (ms)	Log (RMS) log (ms)	Equiv. RR (ms)
Control	Iso 0	Baseline	81.9 ± 1.4	732 ± 13	0.05 ± 0.06	1.13 ± 0.15	0.49 ± 0.04	3.07 ± 0.27
Control	CCh 0	Baseline	81.8 ± 0.9	734 ± 08	0.11 ± 0.05	1.29 ± 0.15	0.57 ± 0.04	3.72 ± 0.33
Ts65Dn	Iso 0	Baseline	79.9 ± 1.4	751 ± 13	−0.11 ± 0.08	0.77 ± 0.15	0.39 ± 0.06	2.45 ± 0.39
Ts65Dn	CCh 0	Baseline	79.8 ± 0.8	752 ± 08	−0.02 ± 0.06	0.95 ± 0.14	0.44 ± 0.07	2.78 ± 0.47
Control	Iso 1	Peak HR	76.2 ± 0.8^‡^	788 ± 08				
Ts65Dn	Iso 1	Peak HR	76.4 ± 0.7^†^	785 ± 07				
Control	Iso 10‐15	Recovery plateau	84.5 ± 0.7*	710 ± 06	−0.42 ± 0.10^‡^	0.38 ± 0.09	0.34 ± 0.06	2.21 ± 0.32
Ts65Dn	Iso 10‐15	Recovery plateau	89.4 ± 1.8*, ‡	671 ± 13	−0.32 ± 0.14	0.48 ± 0.16	0.40 ± 0.06	2.49 ± 0.32
Control	CCh 2‐6	Maximal slowing	147.8 ± 8.0^‡^	406 ± 23	0.30 ± 0.10	2.01 ± 0.47	0.76 ± 0.08^†^	5.73 ± 1.00
Ts65Dn	CCh 2‐6	Maximal slowing	134.7 ± 4.9^‡^	446 ± 17	0.41 ± 0.08^‡^	2.57 ± 0.46	0.70 ± 0.07^‡^	5.06 ± 0.80
Control	CCh 6‐10	Partial recovery	148.6 ± 7.0^*^	404 ± 20	0.26 ± 0.10	1.84 ± 0.43	0.84 ± 0.09^†^	6.93 ± 1.41
Ts65Dn	CCh 6‐10	Partial recovery	125.5 ± 4.5^*^	478 ± 18	0.17 ± 0.09	1.48 ± 0.32	0.67 ± 0.08^†^	4.70 ± 0.87

Note

(Mean group ± standard error of mean)

* Ts65Dn different from control, *p *< .05.

^†^ Different from baseline, *p *< .001.

^‡^ Different from baseline, *p *= .02.

Table 1 lists the group mean values ± the standard error of the mean (*SEM*) for all the parameters, groups, injections, and analysis periods. For averaging periods of minutes or longer, RR‐orig and RR‐vslow are nearly the same, and only RR‐orig is shown here. Just after injection, when the mouse has been out of the cage for approximately 30–60 s, and where the HR is changing rapidly due to drug onset, RR‐orig is more useful than RR‐vslow. Also, during this brief period, the SD‐slow and SD‐fast components are not very useful, thus were not included in the table. In the RR‐orig column, the primary analysis was run on the RRIs, but for convenience of the reader, the equivalent instantaneous HR and *SEM* values were calculated. The RMS(SD‐slow) and RMS(SD‐fast) parameters were log transformed, resulting in a more normal distribution, with more appropriate application of statistical testing. However, since the log parameters are rather abstract, they were back‐calculated into equivalent RR units for the convenience of the reader. Note that RMS of the SD‐fast component is typically two to six times larger than the SD‐slow component.

## 4 DISCUSSION

In this study, we used a new, noninvasive ECG recording technique to analyze heart rate and HRV in the Ts65Dn mouse model of DS and euploid littermate control animals under the effect of an adrenergic and a cholinergic agonist. Based on recent studies relating HRV to various pathologies (Akgul et al., 2019; Nystrom et al., 2019; Roque et al., 2014), and to DS in particular (Carvalho et al., 2018), our driving hypothesis was that Ts65Dn mice would have an alteration in autonomic modulation of HRV when compared with the control euploid animals.

We were able to demonstrate that, even in an animal model such as the Ts65Dn mouse, which displays hyperactive behavior when exposed to new environments, this new recording method generated long stretches of analyzable recordings of heartbeats from which rich HRV can be derived. The performance of our ECG recording apparatus far exceeded that of currently commercially available, noninvasive ECG recording devices. For example, in the past, we worked extensively with the ECGenie, from (Mouse Specifics, Inc. Framingham, MA), but that device requires that the mouse remains still, and thus only collects data for a few seconds at best. Thus, the ECG recording apparatus described in this study is indeed far superior to the ECGenie. The only other noninvasive alternative to obtaining mouse heart rates that we are aware of is through the use of pulse oximeters, such as the Kent Scientific MouseSTAT®. However, to obtain reading from awake mice, the animals need to be immobilized. We have also collected extensive data from an invasive monitor (G2 HR E‐Mitter, STARR Live Sciences, Corp. Oakmont, PA, formerly Mini‐Mitter Co. Inc.), which not only requires the surgical implantation of the device, but can also produce large stretches of recording artifacts.

We chose a mouse model of DS because there are several unresolved issues regarding the origin of HRV differences seen in persons with DS. Although it is true that the use of mouse models provides a unique chance to pharmacologically and genetically dissect genotype‐dependent differences observed in human disorders, it is also true that, in general, phenotypic differences between mouse models of DS and their euploid controls tend to be modest. Therefore, this project presented a great opportunity for us to test the usefulness of the new recording technique we were developing in the laboratory.

Two pharmacological agents were used in this study: Iso and CCh. Isoproterenol is a synthetic catecholamine and belongs to the group of drugs whose metabolic inactivation depends mainly on either catechol‐O‐methyltranferase (COMT), by O‐methylation, or on monoamine oxidases (MAO), by deamination (Rockman, Koch, Milano, & Lefkowitz, 1996). Therefore, not only there was a chance of finding genotype‐dependent differences in sensitivity to this pharmacological agent, but also of generating indirect evidence of potential differences in enzymatic activity between the two groups of animals being investigated.

The second pharmacological agent used here was CCh, which is a synthetic ester of choline and a nonselective cholinergic receptor agonist that acts on all muscarinic and nicotinic receptor subtypes. It is generally accepted that this synthetic ester of choline is not susceptible to acetylcholinesterase action, although its metabolism is not yet fully understood (de La Coussaye et al., 1994).

Data from previous studies showed a link between HRV and the density of beta‐1 adrenergic receptors in the myocardium (Mansier et al., 1996). These phenotypic differences in cardiac muscle have been previously reported in rats, showing that changes in HRV are not only related to the number of autonomic nervous system efferents in the heart, but also to the direct changes in the myocardium in terms of density of their receptors (Carre et al., 1994; Mansier, Chevalier, Barnett, & Swynghedauw, 1993; Mansier et al., 1996). Given some of the reported differences in HRV in individuals with DS, we expected that a similar phenomenon might be at play in Ts65Dn mice, and that we would see genotype‐dependent differences in heart rate and HRV responses to Iso. In addition, we know that the gene encoding the G‐protein‐activated potassium channel subunit 2 (*Girk2*) is located on the Ts65Dn mouse trisomic chromosome 16 segment and, its human homologue (*GIRK2*) is located on chromosome 21. GIRK channels are known to be modulated by a variety of G‐protein‐coupled neurotransmitter receptors (GPCRs) including muscarinic receptors (m2) receptors (Costa, Stasko, Stoffel, & Scott‐Mckean, 2005; Stasko, Scott‐Mckean, & Costa, 2006). Therefore, we expected that its overexpression in Ts65Dn mice might lead to exacerbated effects of CCh on the heart rate and HRV on these mice.

Our results did not fully support either of the hypotheses outlined in the previous paragraph. Although, we found a significant genotype‐dependent difference for the Iso, it was not an effect on the peak positive chronotropic response to this pharmacological agent. Instead, we observed a genotype‐dependent increase in the negative chronotropic rebound effect of this agent in Ts65Dn mice compared with control euploid animals.

Regarding the response to CCh, opposite to what we expected, we found an overall trend toward a smaller response of Ts65Dn versus control euploid mice. The difference, however, only reached significance in the 6–10 min interval after the administration of this agent. Obviously, we would like to capitalize on this difference, and claim to have found evidence of differential cholinergic modulation of heart rate and HRV on Ts65Dn mice. However, it is important to note also that this phenomenon may simply be a result of a significant weight difference between the control euploid mice (34.4 ± 1.82 g; mean ± *SEM*; *n* = 12) and Ts65Dn mice (26.5 ± 1.56 g; *n* = 12; *p* = .0033). It is important to remember, that as a rule of thumb, smaller animals tend to metabolize and excrete pharmacological agents at a faster rate than larger animals. Therefore, more studies (e.g., with subtype‐specific muscarinic agonists, genetic manipulations, and potentially controlled‐release drug infusions) will be necessary for us to gain a greater understanding of the origin of this finding.

## 5 CONCLUSION

The EEG recording and analysis techniques described here were robust and reproducible. Because of the simplicity of the new ECG recording method described here, we believe that this technique will be a welcome addition to the armamentarium of cardiac physiologists working with rodent models of human disorders and diseases. Regarding the overall goal of shedding more light onto mechanistic issues related to heart rate and HRV differences in persons with DS, this study succeeded in proving the principle that such studies can be undertaken with the use of the techniques described here. However, future studies will be necessary to complement the findings in the present work. For example, it will be critical to use more selective pharmacological agents and/or genetic manipulations of the Ts65Dn mouse. Finally, other mouse models of DS may also help us draw a clearer picture of the role of trisomy 21 on generating alterations in heart rate control and on HRV.

## CONFLICT OF INTEREST

None of the authors has any conflict of interest.

## AUTHORS’ CONTRIBUTIONS

A.L.R., M.R.S., and M.W.J.: conception and design, collection and assembly of data, data analysis and interpretation, manuscript writing; L.C.A. and T.D.S.: interpretation and manuscript writing; A.C.S.C.: conception and design, financial support, assembly of data, data analysis and interpretation, manuscript writing, final approval of manuscript.

## Supporting information



Fig S1
Fig S2
