# Supplementary material for: Noninvasive assessment of autonomic modulation of heart rate variability in the Ts65Dn mouse model of Down syndrome: A proof of principle study
Source: Physiol Rep. 2020 Jun 19;8(12):e14486. doi: 10.14814/phy2.14486 (PMC7305244; doi:10.14814/phy2.14486)
Supplement: Supplementary file 1 — Fig S1 Fig S2 [file PHY2-8-e14486-s001.pdf]

Supplementary Information

The ECG detection methods described here worked well to get ECG traces and RRI measures throughout the two-hour recording period, but the yield (estimated percentage of good RRIs measured) varied widely, depending on mouse behavior. The yield estimate was calculated for every 15-second epoch. The yield ranges reported here were taken as the low and high values of the middle 80% of the histogram of yields. For example, from all control mice during the baseline period, 80% of the epochs had good RRI yield between 3% and 27%, 10% of epochs had less than 3% good RRIs, and 10% of epochs had better than 27% good RRIs. By comparison, the Ts65Dn mice had a lower baseline yield of 3-14%, which is not surprising since they tend to display a higher level of exploratory activity in a new cage. In contrast, during the 30-minute period after both Iso and CCh injections, the yield was much higher, ranging from 37-70%, except for the Ts65Dn mice given CCh, which had a lower yield of 20-50%, again because they tended to move around more. Each of the 24 recordings had an estimated 80,000 beats, with typically between 25,000 and 35,000 good RRIs detected. Figure S1 shows the estimated fraction of detected beats for all animals separated by genotype and injection type.

Figure S2 shows an example ECG with of brief sections of poorly detectable beats. This five second sample shows three tiny dropped out sections, but also shows the beginning of a two-second section where approximately 16 beats were missed. This type of signal loss is scattered throughout the recording, and may last for many seconds.

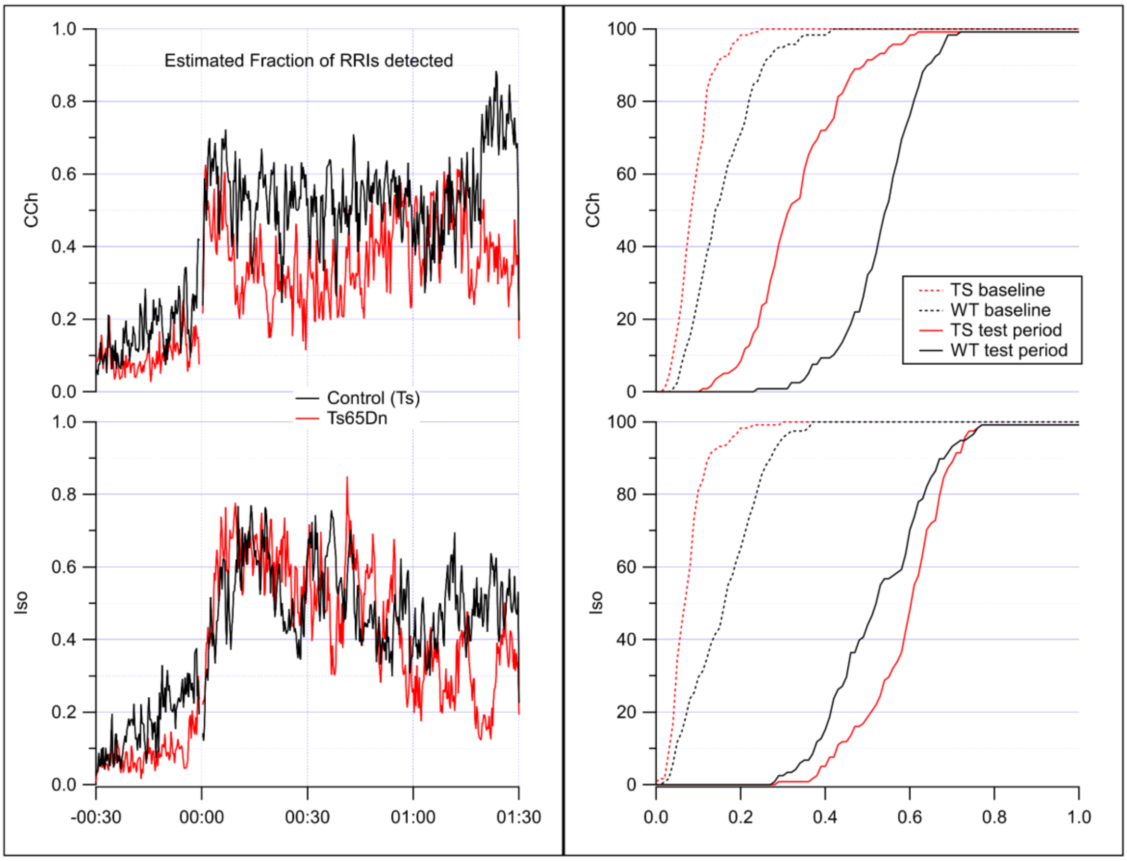

Figure S1. The left-hand panel shows the estimated fraction of detected beats throughout the two-hour studies for all animals. The time scale is elapsed time relative to the injection. The upper and lower sections are for CCh and Iso injections respectively. The expected number of beats in any epoch is the average HR during the epoch times the number of seconds in the epoch. The right panel shows the cumulative histograms of the fraction of RRIs detected during the baseline and test periods. The Ts65Dn mice tend to explore the cage more continuously, so their ECG yield fractions were lower than control mice in both CCh and Iso test sessions. Also, after recovery from the CCh injection the Ts65Dn mice went back to exploring the cage sooner, and again had a lower ECG yield compared to the controls.

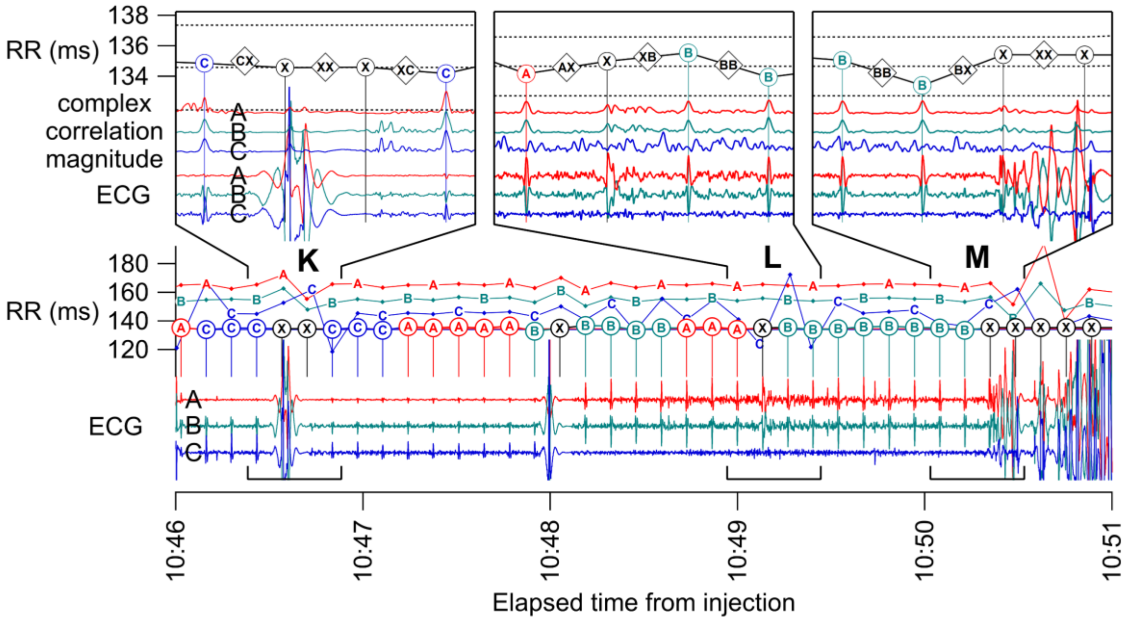

Figure S2. A detailed illustration of the three-channel ECG detection process from a five second segment where mouse movements resulted in automatic switching between channels as well as several brief segments where no channel was acceptable. At the bottom of the graph are the ECG traces for channels A, B and C. Note the abrupt changes in amplitude and shape among the channels. Above the ECG traces are the RRIs separately detected from each channel, and the jointly selected “best” beat times and associated RRIs (circles), with the letters A-C indicating which channel was used, or the letter X when no channel was acceptable. The algorithm projects an approximate time for the missed beat in those cases. The uppermost traces show selected RRIs on a more expanded 0.5 s time scale, with diamond letters indicating the channels of the pair of beats used for the analysis. Note that if either beat is marked X, then the RRI is excluded. Thus one missed beat excludes two RRIs. The three traces just above K-M are the ECG traces on the expanded scale. The three traces above that are the complex correlation magnitude, which are the primary metric used to compare channels and select the best channel. K shows large amplitude movement artifact and amplifier recovery (flat section). L shows muscle artifact resulting in one missed beat. M shows the beginning of a period of sustained movement.
